# Ser253Leu substitution in PmrB contributes to colistin resistance in clinical *Acinetobacter nosocomialis*

**DOI:** 10.1080/22221751.2021.1976080

**Published:** 2021-09-17

**Authors:** Ya-Sung Yang, Wen-Yih Jeng, Yi-Tzu Lee, Chi-Ju Hsu, Yu-Ching Chou, Shu-Chen Kuo, Cheng-Cheung Chen, Wei-Jane Hsu, the ACTION study group, Hsing-Yu Chen, Jun-Ren Sun

**Affiliations:** a Division of Infectious Diseases and Tropical Medicine, Department of Internal Medicine, Tri-Service General Hospital, National Defense Medical Center, Taipei, Taiwan; b University Center for Bioscience and Biotechnology, National Cheng Kung University, Tainan, Taiwan; c Department of Biochemistry and Molecular Biology, National Cheng Kung University, Tainan, Taiwan; d School of Medicine, National Yang-Ming University, Taipei, Taiwan; e Department of Emergency Medicine, Taipei Veterans General Hospital, Taipei, Taiwan; f Institute of Preventive Medicine, National Defense Medical Center, Taipei, Taiwan; g School of Public Health, National Defense Medical Center, Taipei, Taiwan; h National Institute of Infectious Diseases and Vaccinology, National Health Research Institutes, Zhunan, Taiwan; i Graduate Institute of Medical Sciences, National Defence Medical Centre, Taipei, Taiwan; j Department of Medical Techniques, Taipei City Hospital Ren-Ai Branch, Taipei, Taiwan

**Keywords:** Colistin resistance, Acinetobacter nosocomialis, pmrB, MLST, pmrCAB operon

## Abstract

Infections caused by extensively drug-resistant (XDR) *Acinetobacter nosocomialis* have become a challenging problem. The frequent use of colistin as the last resort drug for XDR bacteria has led to the emergence of colistin-resistant *A. nosocomialis* (ColRAN) in hospitals. The mechanism of colistin resistance in *A. nosocomialis* remains unclear. This study aimed to investigate the mechanisms underlying colistin resistance in clinical ColRAN isolates. We collected 36 *A. nosocomialis* isolates from clinical blood cultures, including 24 ColRAN and 12 colistin-susceptible *A. nosocomialis* (ColSAN). The 24 ColRAN isolates clustered with ST1272 (13), ST433 (eight), ST1275 (two), and ST410 (one) by multilocus sequence typing. There was a positive relationship between *pmrCAB* operon expression and colistin resistance. Further analysis showed that colistin resistance was related to an amino acid substitution, Ser253Leu in PmrB. By introducing a series of recombinant PmrB constructs into a PmrB knockout strain and protein structural model analyses, we demonstrated that the association between Ser253Leu and Leu244 in PmrB was coupled with colistin resistance in ColRAN. To the best of our knowledge, this is the first study demonstrating that the key amino acid Ser253Leu in PmrB is associated with overexpression of the *pmrCAB* operon and hence colistin resistance. This study provides insight into the mechanism of colistin resistance in *A. nosocomialis*.

## Introduction

A unneglectable number of extensively drug-resistant (XDR) *Acinetobacter* spp. that have acquired resistance to multiple classes of antibiotics has emerged [1–3]. XDR *Acinetobacter* spp. are resistant to nearly all antimicrobial agents, including carbapenems, cephalosporins, penicillins, aminoglycosides, and fluoroquinolones [4]. In the absence of antibiotic selection, colistin has become a very limited option [5]. In current medical practice, colistin is becoming more frequently prescribed to treat XDR *Acinetobacter* spp. However, the increased use of colistin has led to the development of colistin resistance in such pathogens [6,7]. *Acinetobacter baumannii* and *A. nosocomialis* are the two most frequently observed *Acinetobacter* clinical isolates [3,8,9]. *A. nosocomialis* has a higher rate of colistin resistance than *A. baumannii* [10,11] and the difference is even greater when both species are carbapenem-resistant (45.3% vs. 1.4%) [11–13].

The two main colistin-resistance mechanisms have previously been described in *A. baumannii*. The first mechanism is the modification of the lipid A component in LPS mediated by the *pmrCAB* operon. The expression of the *pmrCAB* operon is tightly regulated by the PmrA/PmrB two-component system, which contains a sensor kinase (PmrB) and a response regulator (PmrA) encoded by the same *pmrCAB* operon. Once the PmrA/PmrB two-component system is activated, phosphorylated PmrA binds to the promoter region of the *pmrCAB* operon and stimulates the expression of the *pmrCAB* operon to generate lipid A phosphoethanolamine transferase, which leads to the modification of lipid A [14,15]. This modification reduces the net negative charge of the outer membrane and results in colistin resistance [16]. The second mechanism is the complete loss of LPS expression to impaired lipid A synthesis via mutations in the *lpxA*, *lpxC*, and *lpxD* genes [17–19]. Recently, plasmid-mediated colistin resistance (*mcr*) genes have been found to be associated with colistin resistance [9].

In the case of other *Acinetobacter* spp., studies regarding the mechanisms of colistin resistance remain limited. In the current study, we focused on the mechanism of resistance in clinical colistin-resistant *A. nosocomialis* (ColRAN) blood isolates.

## Materials and methods

### Bacterial strains and antimicrobial susceptibility testing

Isolates of *A. nosocomialis* were collected from clinical blood samples at four medical centres of AntimiCrobial studies in Taiwan Operating Network (ACTION) from 2009 to 2015. The medical centres included Changhua Christian Hospital (CCH), Mackay Memorial Hospital (MKH), Taipei Veterans General Hospital (TVGH), and Tri-Service General Hospital (TSGH) of the National Defense Medical Center (NDMC). This study was approved by the Ethics Committee of the aforementioned centres. The minimum inhibitory concentration (MIC) of colistin was evaluated using the broth microdilution method [20]. Colistin resistance was defined as the presence of an MIC >2 mg/L.

### Molecular typing

The clonal relationships of the isolates were determined using multilocus sequence typing (MLST) and pulsed-field gel electrophoresis (PFGE) [11]. All isolates subjected to PFGE were analysed using BioNumerics software (Applied Maths) to determine the relatedness of the isolates. Isolates were considered to be of different types if they had more than three DNA fragment differences and a similarity of <80% in dendrogram analysis. MLST was performed using Pasteur schemes, as suggested in a previous study [21]. Allelic profiles (in the order: cpn60-fusA-gltA-pyrG-recA-rplB-rpoB) were used to assign sequence types (STs) to all isolates. eBURST analysis (http://eburst.mlst.net) was performed to assign each ST to a respective clonal complex (CC) [22].

### Polymerase chain reaction (PCR) amplification and DNA sequencing

To detect the plasmid-mediated colistin-resistance gene, multiplex PCR was performed to screen *mcr-1*, *mcr-2*, *mcr-3*, *mcr-4*, and *mcr-5* genes, as described previously [23]. The genetic loci implicated in colistin resistance were amplified using PCR and sequenced, including the *pmrCAB* operon, *lpxAD* region, and *lpxC* gene (Supplementary Table 1). Nucleotide sequences of *pmrB* genes from Type 1 to Type 6 were submitted to the GenBank database and assigned accession numbers MW241539, MW241540, MW241541, MW241542, MW241543, and MW241544, respectively.

### Transcriptional analyses by quantitative real-time PCR assays

The transcription levels of *pmrC*, *pmrA*, *lpxA*, and *lpxC* genes were measured by quantitative real-time PCR assays. Real-time PCR was performed using the QuantiNova SYBR Green PCR Kit (Qiagen, Hilden, Germany). mRNA of the *rpoB* gene was used as a control and ATCC17903 was used as a reference to the standard expression level.

### Mutants of pmrb deletion and expression experiments

The bacterial strains and plasmids used in this study are listed in Supplementary Table 2. ATCC17903Δ*pmrB* was an unmarked deletion mutant created as suggested in a previous study [24]. *pmrB* gene fragments were cloned and generated into a series of recombinant pS01_*pmrB* clones (ATCC17903, Type 1, and Type 2). Specific nucleotide mutations in the *pmrB* gene were generated using overlap extension PCR. The mutated *pmrB* genes were cloned to produce pS01_*pmrB* (Type 2, Ile243Ser) and pS01_*pmrB* (Type 2, Leu244Ser).

### Structural modelling analysis

The structural model of wild-type *A. nosocomialis* PmrB (Type 2) was constructed using SWISS-MODEL [25,26], a fully automated protein structure homology-modelling server, using the Protein Data Bank accession code 4BIV (the cytoplasmic region of *Escherichia coli* sensor histidine kinase CpxA) as the template [27]. The structural model of the *A. nosocomialis* PmrB Ser253Leu mutant was generated by Coot using the wild-type PmrB modelling structure [28]. Structural figures were produced using PyMOL (DeLano Scientific, http://www.pymol.org).

### Data analysis

Statistical analysis was performed using SPSS version 20.0, and statistical differences among various groups were calculated using the Mann–Whitney test. Differences were considered statistically significant at *p* <0.05.

## Results

### MLST and PFGE profiles among ColRAN isolates

Twenty-four ColRAN isolates were collected and classified into four STs using the Pasteur MLST scheme as follows: ST1272, ST433, ST1275, and ST410. The largest cluster was ST1272 (83-26-79-14-27-16-47) with 13 isolates, followed by ST433 (22-26-29-14-27-16-47), ST1275 (22-26-54-14-191-16-118), and ST410 (20-26-26-14-26-16-23), with eight, two, and one isolate, respectively. There were at least two mismatches in the seven loci between the four STs. e-BURST analysis identified three independent CCs, namely CC410 (ST410), CC1272 (ST1272), CC782 (ST433), and ST1275 as a singleton. None of the CCs were related to each other after the analysis. The MICs of ColRAN isolates ranged from 4 to 16 mg/L. Using multiplex PCR, none of the isolates were found to have detectable *mcr-1*, *mcr-2*, *mcr-3*, *mcr-4*, and *mcr-5* genes. To study the mechanism of colistin resistance, we randomly selected eight ColSAN isolates with the following MLST types as the comparison groups: ST1272 (two isolates), ST433 (three isolates), and ST410 (three isolates) from the ACTION group. For ColSAN isolates, the MICs of colistin ranged from 0.5–2 mg/L. The 24 ColRAN and eight ColSAN isolates were classified into six clusters (28 isolates, 87.5%) and four unique PFGE types based on the dendrogram (Supplementary Figure 1).

### Transcription level of the pmrCAB and lpxACD operons

**Figure 1.**
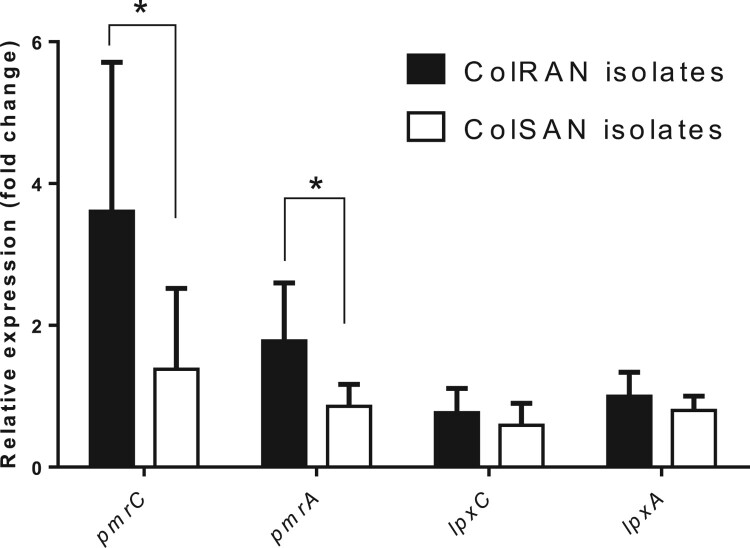
Relative expression of *pmrC*, *pmrA*, *lpxC,* and *lpxA* genes in *Acinetobacter nosocomialis* isolates. Each isolate was tested in triplicate in two independent experiments. Bars represent the average, and error bars represent standard deviations. Black bars, colistin-resistant *Acinetobacter nosocomialis* (ColRAN) isolates; white bars, colistin-susceptible *Acinetobacter nosocomialis* (ColSAN) isolates. Data were analysed using an independent t-test (*p <0.05).

Comparing all ColSAN and ColRAN isolates, the RNA expression levels of the *pmrC*, *pmrA*, *lpxC*, and *lpxA* genes of the ColRAN isolates were 2.6-, 2.1-, 1.3-, and 1.3-fold higher than those of the ColSAN isolates, respectively (Figure 1). The transcription levels of the *pmrC* and *pmrA* genes in ColRAN isolates were significantly higher than those in ColSAN isolates (both *p* < 0.05). However, neither *lpxC* nor the *lpxA* gene was overexpressed.

### The association of PmrAB two-component systems and colistin resistance

Amino acid analysis revealed that the isolates possessed three types of PmrA (A, B, and C types), and six types of PmrB (Types 1–6), including one novel variant (Type 3) (Supplementary Table 3). There was a significant difference in the PmrB pattern between the ColRAN and ColSAN isolates (*p* < 0.05). The most common combination of the PmrA-PmrB pattern was A-2 (19 isolates), with colistin MICs ranging from 4 to 8 mg/L (Table 1). Among the 19 ColRAN isolates with the A-2 pattern, 16 had *pmrC* overexpression (84.2%). Therefore, we believe that colistin resistance mediated by *pmrC* overexpression may be associated with amino acid substitutions in the PmrB sensor sequence (Type 2).

**Table 1.** Pasteur multi-locus sequence typing profile and amino acid variation patterns in PmrA and PmrB proteins.

	Colistin MIC (mg/L) PmrA–PmrB pattern (isolates)					
MLST type (n)	0.5	1	2	4	8	16
ST1272 (15)		C-4 (1)	C-4 (1)	A-2 (6) C-4 (1)	A-2 (6)	
ST433 (11)	A-1 (1)	A-1 (2)		A-2 (7)		A-3 (1)
ST410 (4)	A-5 (1)	A-5 (2)		A-5 (1)		
ST1275 (2)				B-6 (2)		

Abbreviations: MIC, minimum inhibitory concentration; MLST, multi-locus sequence typing; ST, sequence type.

### Analysis of amino acid substitutions within the PmrB sensor

After comparison with the reference strain ATCC17903, 15 amino acid substitutions were found in the six PmrB sequences (Supplementary Table 4). The major types of PmrB were Type 2, including ST1272 (12 isolates) and ST433 (seven isolates). There was a distinctive amino acid substitution (Ser253Leu) between type 1 and type 2 PmrB. In comparison to Type 1, the colistin MIC values of Type 2 showed an 8-fold increase. Among the clinical isolates, the transcription level of *pmrC* in isolates carrying Type 2 PmrB was significantly higher than that in isolates carrying Type 1 PmrB (3.63 ± 1.8 vs. 0.87 ± 0.76, *p* < 0.05). Hence, it was necessary to investigate whether colistin resistance was associated with this amino acid substitution in PmrB.

### Complementation of PmrB mutations

The MIC of colistin against ATCC17903Δ*pmrB* carrying pS01_*pmrB* (ATCC17903) was higher than that of the strain carrying pS01 (empty vector) and was associated with a higher expression level of the *pmrC* gene (Table 2). ATCC17903Δ*pmrB* transformed with pS01_*pmrB* (Type 2) showed a higher MIC of colistin than pS01_*pmrB* (Type 1) (16 vs. 2 mg/L). The transcription levels of the *pmrC* and *pmrA* genes in ATCC17903Δ*pmrB* with pS01_*pmrB* (Type 2) were significantly higher than those in pS01_*pmrB* (Type 1). Furthermore, the transcription levels of *lpxA* and *lpxC* in the two transformants were not significantly different. Hence, the Ser253Leu point substitution in PmrB appeared to be the critical amino acid substitution causing colistin resistance.

**Table 2.** Relationship between colistin minimum inhibitory concentrations and expression of *pmrC*, *pmrA*, *lpxC*, and *lpxA* genes in various transformants.

			Differential quantification of gene expression (fold change)			
Name	with plasmid	Colistin MIC (mg/L)	*pmrC*	*pmrA*	*lpxC*	*lpxA*
ATCC17903 (wt)	no plasmid	16	19 (7.1)	14.4 (2.5)	1.2 (0.2)	1.0 (0.2)
ATCC17903Δ*pmrB*	no plasmid	1	1.0	1.0	1.0	1.0
	pS01 (vector only)	1	1.2 (0.1)	1.5 (0.3)	1.2 (0.2)	0.9 (0.2)
	pS01_*pmrB* (ATCC17903)	16	44.5 (6.6)	17.1 (2.9)	0.9 (0.1)	1 (0.1)
	pS01_*pmrB* (Type 1)	2	4.3 (0.6)	3.3 (0.6)	1.3 (0.3)	1.0 (0.3)
	pS01_*pmrB* (Type 2)	16	29.8 (5.7)	13.3 (3.1)	0.9 (0.2)	0.9 (0.1)
	pS01_*pmrB* (Type 2, Ile243Ser)	16	47.3 (12.1)	5.9 (2.0)	0.9 (0.2)	1.0 (0.2)
	pS01_*pmrB* (Type 2, Leu244Ser)	2	7.7 (1.1)	1.4 (0.3)	1.2 (0.2)	1.4 (0.2)

Abbreviations: MIC, minimum inhibitory concentration.

### Structural analysis of PmrB with critical ser253leu substitution

**Figure 2.**
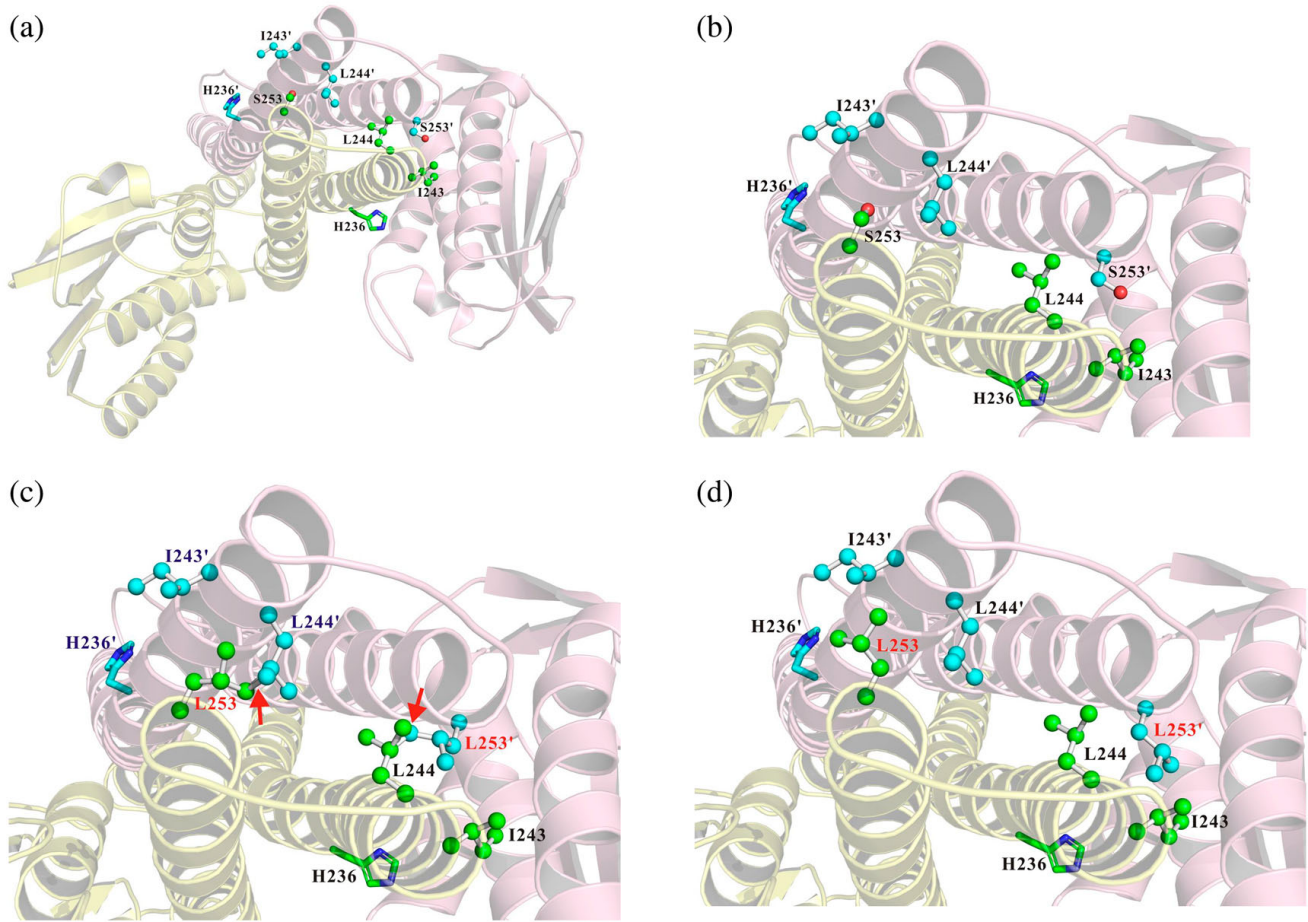
Structural models of PmrB. (a) The modelling structure of the Type 1 PmrB homodimer is shown as a cartoon diagram; one subunit is shown in light pink and the other is shown in light yellow. (b, c, and d) Close-up view of two types of orientations of Ser253Leu (Type 2a and Type 2b) with substitution of PmrB. The side chains of His236 and His236' are shown as green and cyan stick models, respectively. The carbon atoms of Ile243, Leu244, Ser253, and L253 are shown as blue ball and stick models, respectively. The carbon atoms of Ile243', Leu244', Ser253', and L253' are shown as cyan ball and stick models. Oxygen atoms are shown in red. The steric collisions between L253 and L244' or L253' and L244 of Type 2b are indicated by arrows (b).

To address the structural effect of PmrB with the critical amino acid substitution of Ser253Leu on colistin resistance, a structural model of the Type 1 PmrB homodimer was generated by the homology-modelling server using the CpxA structure as the template (Figure 2a and b). The Ser253Leu substitution of PmrB resulted in two possible orientations of the side chain of the Leu253 residue. In the first orientation, the side chain of Leu253 was too close to the side chain of Leu244 on the opposite subunit of the PmrB homodimer, causing a steric collision (Figure 2c). In the second orientation, the side chain of Leu253 was close to the side chain of Ile243 on the opposite subunit of the PmrB homodimer, increasing hydrophobic interactions between these two residues (Figure 2d).

To identify the critical amino acid interacting with Leu253, two types of mutations (Ile243Ser and Leu244Ser) were constructed in Type 2 PmrB. ATCC17903Δ*pmrB* transformed with pS01_*pmrB* (Type 2, Leu244Ser) showed significantly lower MICs of colistin than pS01_*pmrB* (Type 2) (2 vs. 16 mg/L) (Table 2). Comparing the two transformants, the transcription levels of the *pmrC* and *pmrA* genes were related to the MIC of colistin. Summarizing the results of the structural modelling and MIC assays, Leu253 in PmrB was possibly related to colistin resistance by pushing Leu244 on the opposite subunit of the PmrB homodimer.

## Discussion

**Figure 3.**
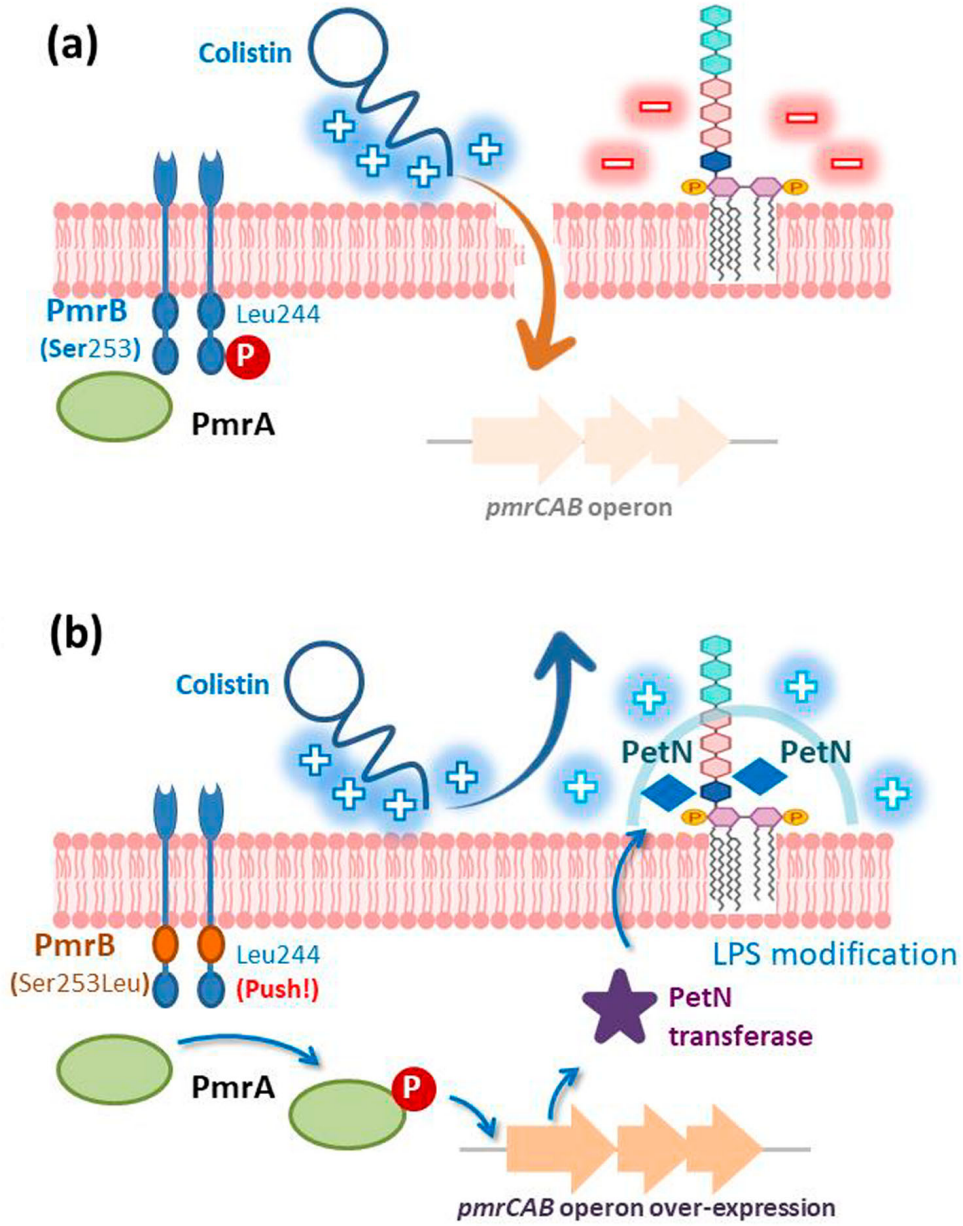
Schematic representation for the mechanism of colistin resistance. (a) Colistin is a cationic antimicrobial peptide. Colistin targets the anionic lipid A portion of lipopolysaccharides (LPS) and binds to phospholipids in bacterial cell membranes. This binding leads to changes in the permeability of the outer cell membrane and leakage of cell contents. The PmrAB locus is a two-component system (TCS) that can regulate the expression of the *pmrCAB* operon. The *pmrCAB* operon usually has a low expression level and encodes three functional proteins, including PmrC (PetN transferase), PmrA (response regulator), and PmrB (sensor kinase). (b) A model based on our data: amino acid substitution in PmrB (Ser253Leu) caused overexpression of the *pmrCAB* operon. Overexpression of the *pmrC* gene will generate several phosphoethanolamine (PetN) transferases. PetN transferase can add PetN to either the 4' or 1' phosphate of lipid A in LPS. This modification of LPS results in positively charged phosphate groups and prevents the binding of the cationic colistin.

The emergence of colistin resistance in *Acinetobacter* spp. is a serious clinical threat. However, an in-depth investigation of the mechanism of colistin resistance in ColRAN is still lacking. This is the first detailed description of the mechanism of resistance caused by an amino acid substitution (Ser253Leu) in PmrB. Under normal circumstances, colistin targets the anionic lipid A portion of LPS and binds to phospholipids in bacterial cell membranes, leading to changes in the permeability of the outer cell membrane and leakage of cell contents (Figure 3a). The Ser253Leu substitution in this study was further identified to increase the interaction with Leu244 on the opposite side of PmrB and promote conformational changes in PmrB dimers. This interaction within PmrB drives the expression of the *pmrCAB* operon via PmrA phosphorylation. The PetN transferase encoded by the *pmrC* gene increases and adds the positively charged PetN to lipid A. Finally, the positively charged lipid A of LPS prevented the binding of cationic colistin (Figure 3b).

Among the ColRAN isolates found in this study, 12 isolates (54.2%) belonged to ST1272, and eight isolates (33.3%) belonged to ST433. We traced the source of our isolates by searching the MLST database and found that ST1272 is a novel ST identified in this study. ST433 was originally isolated in Taiwan in 2003. In contrast, ST410 was found to be the most dominant type of clinical carbapenem-resistant (CR) *A. nosocomialis* isolates in Taiwan [29]. Notably, in our unpublished study analysing the correlation between MLST types and carbapenem resistance, we found that the carbapenem resistance rates among different MLST types of *A. nosocomialis* isolates were 4.8, 100, and 46.7% for ST410, ST433, and ST1272, respectively. These results suggest that *A. nosocomialis*, with different MLST types under antibiotic stress, has different evolutionary capabilities. Unknown fitness advantages exist in ST1272 and ST433, and these advantages probably drive them to develop colistin resistance in the hospital setting and under antimicrobial selection pressure [6,7,13].

In this study, six ColRAN isolates (MIC = 4–8 mg/L) carried Type 2 PmrB (three), Type 5 PmrB (one), and Type 6 PmrB (two), and did not show overexpression of *pmrC*. Some unidentified factors may have affected the expression of *pmrCAB* in these clinical isolates. Among the three isolates carrying type 2 PmrB, without overexpressed *pmrC*, colistin resistance was probably due to mechanisms other than *pmrCAB* activation. Previous studies have identified that other genetic determinants conferred colistin resistance in *A. baumannii*, including chromosome-mediated mechanisms (the *eptA* gene) and plasmid-mediated mechanisms (*mcr* genes) [30,31]. To identify the cause of colistin resistance, we also performed whole genome sequencing of these ColRAN isolates and found no relevant mechanism (data not shown). Further in-depth investigations are required to elucidate the mechanism of colistin resistance in these isolates.

In the PmrB Ser253Leu mutant model, Leu244 and Ser253/Leu253 residues were located on the respective alpha-helices of dimerization and the histidine phosphotransfer (DHp) domain. The side chain of the Leu253 residue was too close to the Leu244 residue on the opposite subunit of the PmrB homodimer, which then pushes out the DHp domain and causes helical bending of the histidine kinase DHp domain. In previous reports focused on the *A. baumannii* two-component system-mediated regulation of the AdeRS two-component system, the helical bending of the histidine kinase DHp domain, caused by a highly conserved proline residue (Pro154 of AdeS) in the proximity of phosphorylated histidine residues (His149 of AdeS), was determined to be essential for histidine kinase activation [27,32,33]. Combining the results of structural modelling and MIC assays, we speculated that Leu253 residues present a van der Waals repulsive force to the Leu244 residue in the PmrB Ser253Leu mutant, resulting in the constitutive helical bending conformation of the PmrB DHp domain, leading to the activation of PmrB and hence colistin resistance.

## Conclusion

To the best of our knowledge, this is the first study to identify a new Ser253Leu substitution in PmrB and mediate colistin resistance in clinical *A. nosocomialis* isolates. The effect of this substitution in the PmrB-activated *pmrCAB* operon created by the potential association between these two amino acids (Leu253 and Leu244) led to colistin resistance. The substitution position of Ser253Leu is key for a deeper understanding of the mechanism of colistin resistance in ColRAN isolates. The discovery of this result provided an in-depth investigation of the mechanism of colistin resistance and shed light on drug development for fighting such pathogens.

## Supplementary Material

Clean_copy_of_supplementary_material.docx
