# Supplementary material for: Ser253Leu substitution in PmrB contributes to colistin resistance in clinical Acinetobacter nosocomialis
Source: Emerg Microbes Infect. 2021 Sep 17;10(1):1873–80. doi: 10.1080/22221751.2021.1976080 (PMC8451652; doi:10.1080/22221751.2021.1976080)

Supplementary Table 1.

Oligonucleotides used in this study.

| Primer name | Sequence (5’to 3’) | Features/purpose |
| --- | --- | --- |
| An_pmrB_F (BamH1) | GACGGATCCAGAAGGAGATATACATATGAACGTGCATTATTCATTA | Cloning *pmrB* gene with engineered BamHI site into pS01 to form pS01_*pmrB*. |
| An_pmrB_R (BamH1) | GCAGGATCCGGTTGTTTGGGCAGTGAACATT |  |
| PmrCAB_F (An) | GCTAAGTGAGTCCGAGATTCATATG | *pmrCAB* operon PCR and sequencing |
| PmrCAB_R (An) | TCGTCTTGAACAACCAAAACG |  |
| lpxA_F | GGCACCTCGAGCACTCTATC | *lpxAD r*egion PCR and sequencing |
| lpxD_R | CAAGCTGCTGAGCAATTACG |  |
| LpxC_F | CAGCTGATGCAGAACCAAGA | *lpxC* region PCR and sequencing |
| LpxC_R | GAACAATGGCAGGTTGTGTG |  |
| An_rpoB_RT_F | CTAATGGCGGTGGTTCAACT | RT- PCR of *rpoB* gene |
| An_rpoB_RT_R | ATTTCTGCGCTCTTCTCTTTC |  |
| An_pmrC_RT_F | GCCATGTATGTTCTCTGGTA | RT- PCR of *pmrC* gene |
| An_pmrC_RT_R | ATCCAAGTCACTTGATAGCC |  |
| An_pmrA_RT_F | TGCAAAACCGTGTTGATGG | RT- PCR of *pmrA* gene |
| An_pmrA_RT_R | GCAAGTTGAGCCTCTAC |  |
| An_lpxA_RT_F | GTAGGTGGTTTTACCAGAAT | RT- PCR of *lpxA* gene |
| An_lpxA_RT_R | TATGTAAGCTGCAATGTTCGC |  |
| An_lpxC_RT_F | TGTGGTAAAAGCGAGTGGAATA | RT- PCR of *lpxC* gene |
| An_lpxC_RT_R | GCAATGCATTAGCAGGAATATC |  |
| pmrCAB_Up_F (Pst1) | ACTCTGCAGGGTGGAATGGGTCAATAACG | PCR of 0.6 kb up fragment of *pmrB* region |
| pmrCAB_Up_R (BamH1) | CAGGATCCCGTTCATGAAGTCCCGAAAT |  |
| pmrCAB_Dw_F (BamH1) | TCGGGACTTCATGAACGGGATCCTGGTTTTTCGAACTCAAAGCGATG | PCR of 0.6 kb down fragment of *pmrB* region |
| pmrCAB_Dw_R (Sph1) | CAAGCATGCGTATTCCCAGTGGGTGGAAAA |  |
| An_pmrB (Ile243Ser)_F | AAGAGTTTGTTAAGCCAGTTTCCTGAG | Construction of pS01_*pmrB* (Type 2, I245S) |
| An_pmrB (Ile243Ser)_R | AGGAAACTGGCTTAACAAACTCTTGGTTTGTAAGTTCAATGC |  |
| An_pmrB (Leu244Ser)_F | AAGATTAGTTTAAGCCAGTTTCCTGAG | Construction of pS01_*pmrB* (Type 2, L246S) |
| An_pmrB (Leu244Ser)_R | AGGAAACTGGCTTAAACTAATCTTGGTTTGTAAGTTCAATGC |  |

Supplementary Table 2.

Bacterial strains and plasmids used in this study.

| Strains or plasmids | Relevant characteristics |
| --- | --- |
| ***Acinetobacter nosocomialis* strains** |  |
| ATCC17903 (wt) | *A. nosocomialis* reference strain ATCC17903 |
| ATCC17903Δ*pmrB* | Derived from ATCC17903. *pmrB* deletion |
| Tvgh0278 | clinical MDR strain with *pmrB* (Type 1), Colistin MIC=1 mg/L |
| Tvgh0390 | clinical MDR strain with *pmrB* (Type 2), Colistin MIC=4 mg/L |
| Tvgh0661 | clinical MDR strain with *pmrB* (Type 3), Colistin MIC=16 mg/L |
| Tvgh2309 | clinical MDR strain with *pmrB* (Type 4), Colistin MIC=4 mg/L |
| **Plasmids** |  |
| pS01 | *E. coli*-*Acinetobacter* shuttle plasmid; mini-CTX::lacIq-PT7-*lacZ* |
| pS01_*pmrB* (ATCC17903) | mini-CTX:: Mini-CTX::lacI^q^-P_T7_-*pmrB*-*lacZ*  (*pmrB* from ATCC 17903) |
| pS01_*pmrB* (Type 1) | mini-CTX:: Mini-CTX::lacI^q^-P_T7_-*pmrB*-*lacZ*  (*pmrB* from Tvgh0278) |
| pS01_*pmrB* (Type 2) | mini-CTX:: Mini-CTX::lacI^q^-P_T7_-*pmrB*-*lacZ*  (*pmrB* from Tvgh0390) |
| pS01_*pmrB* (Type 2, Ile243Ser) | Derived from pS01_*pmrB* (Type 2), point mutation in *pmrB* lle243Ser |
| pS01_*pmrB* (Type 2, Leu244Ser) | Derived from pS01_*pmrB* (Type 2), point mutation in *pmrB* Leu244Ser |

Abbreviations: MDR, multi-drug resistant; MIC, minimum inhibitory concentration.

Supplementary Table 3.

The type of PmrA and PmrB proteins in colistin-susceptible and -resistant *Acinetobacter nosocomialis* ColSAN and ColRAN isolates.

|  |  | ColRAN (n=24) | ColSAN (n=8) |  |
| --- | --- | --- | --- | --- |
| Protein pattern | Type  (sequence ID) | N (%) | N (%) | *p* value^a^ |
| PmrA | A_type (WP_002051287) | 21 (87.5) | 6 (75.0) | 0.198 |
|  | B_type  (WP_004746055) | 2 (8.3) | 0 (0) |  |
|  | C_type  (WP_004705846) | 1 (4.2) | 2 (25.0) |  |
| PmrB | Type 1  (WP_004712203) | 0 (0) | 3 (37.5) | <0.001 |
|  | Type 2  (AZC10767) | 19 (79.2) | 0 (0) |  |
|  | Type 3  (MW241541) | 1 (4.2) | 0 (0) |  |
|  | Type 4  (WP_077168769) | 1 (4.2) | 2 (25.0) |  |
|  | Type 5  (WP_006580391) | 1 (4.2) | 3 (37.5) |  |
|  | Type 6  (WP_025469480) | 2 (8.3) | 0 (0) |  |

^a^By Fisher’s exact test

Abbreviations: ColSAN, colistin-susceptible *Acinetobacter nosocomialis*; ColRAN, colistin-resistant *A. nosocomialis.*

Supplementary Table 4.

Amino acid substitutions in PmrB proteins.

|  |  |  | PmrB protein (444 amino acids) | | | | | | | | | | | | | | |
| --- | --- | --- | --- | --- | --- | --- | --- | --- | --- | --- | --- | --- | --- | --- | --- | --- | --- |
| PmrB pattern | MLST type  (isolates) | Colistin  MIC range (mg/L) | 58  Thr | 93  Phe | 99  Gln | 145  Ile | 193  Asp | 196  Leu | 253  **Ser** | 255  Gln | 266  His | 286  His | 363  Phe | 380  Tyr | 417  Thr | 439  Leu | 440  His |
| Type 1 (3) | ST433 (3) | 0.5-1 | - | - | - | Met | - | - | - | - | - | Pro | - | - | - | - | - |
| Type 2 (19) | ST433 (7)  ST1272 (12) | 4-8 | - | - | - | Met | - | - | Leu | - | - | Pro | - | - | - | - | - |
| Type 3 (1) | ST433 (1) | 16 | - | - | - | Met | - | - | - | - | Tyr | Pro | - | Asn | - | - | - |
| Type 4 (3) | ST1272 (3) | 1-4 | Ala | Leu | Glu | Met | Asn | Gln | - | His | - | Pro | Tyr | - | Ser | Ile | Asn |
| Type 5 (4) | ST410 (4) | 0.5-4 | - | Leu | - | Met | - | Gln | - | - | - | Pro | - | - | - | - | - |
| Type 6 (2) | ST1275 (2) | 4 | - | - | - | Met | - | Gln | - | - | - | Pro | - | - | - | - | - |

Abbreviations: MIC, minimum inhibitory concentration.

Supplementary Figure 1.

Pulsed-field gel electrophoresis of 32 *Acinetobacter nosocomialis* isolates.


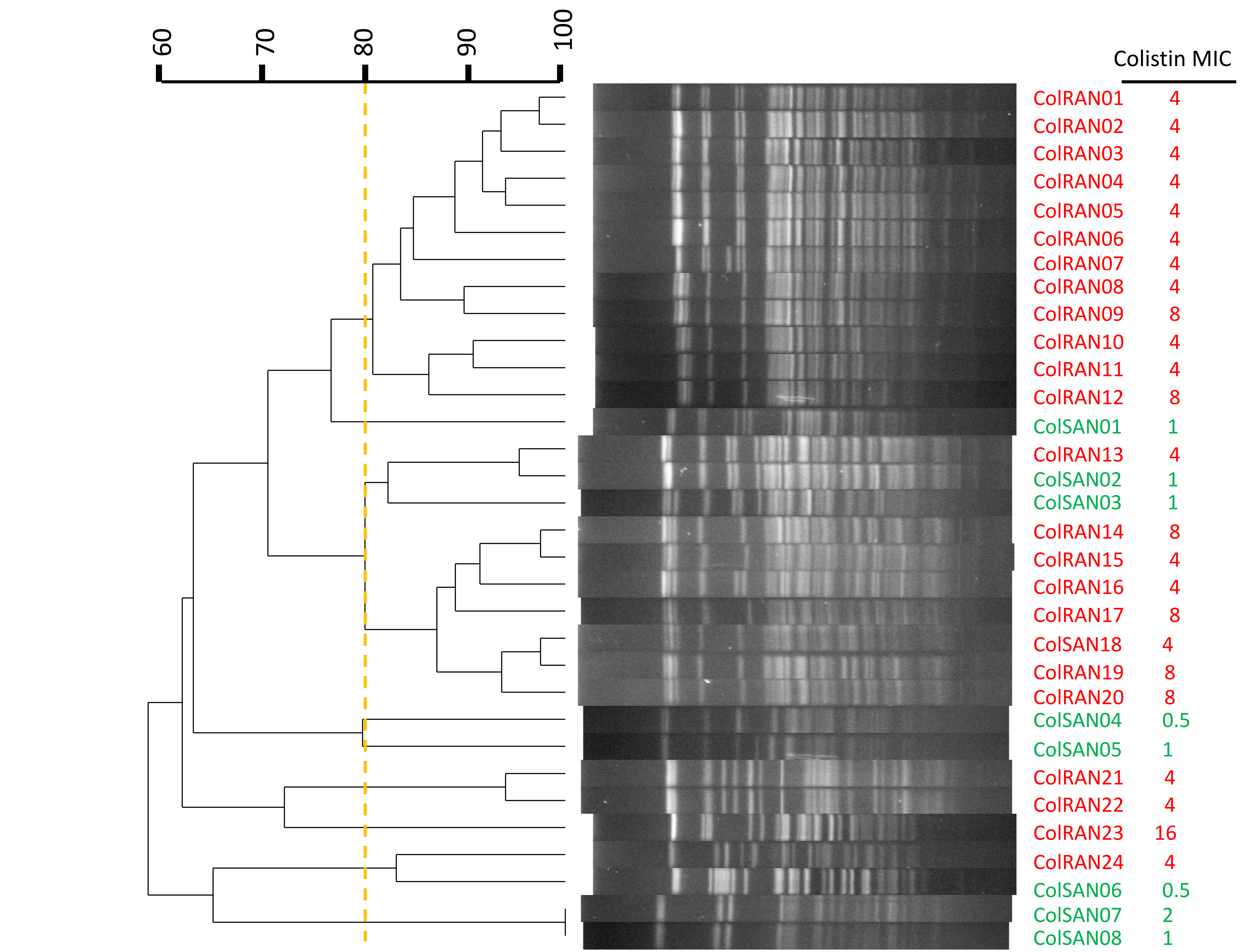

Supplement: Clean_copy_of_supplementary_material.docx [file TEMI_A_1976080_SM1776.docx]
